# Gene surgery: Potential applications for human diseases

**DOI:** 10.17179/excli2019-1833

**Published:** 2019-10-11

**Authors:** Ayman El-Kenawy, Bachir Benarba, Adriana Freitas Neves, Thaise Gonçalves de Araujo, Bee Ling Tan, Adel Gouri

**Affiliations:** 1 Department of Pathology, College of Medicine, Taif University, Saudi Arabia; 2 Department of Molecular Biology, GEBRI, University of Sadat City, P.O. Box 79, Sadat City, Egypt; 3 Laboratory Research on Biological Systems and Geomatics, Faculty of Nature and Life Sciences, University of Mascara, Algeria; 4 Institute of Biotechnology, Molecular Biology Laboratory, Universidade Federal de Goias, Catalao, Brazil; 5 Laboratory of Genetics and Biotechnology, Institute of Biotechnology, Federal University of Uberlandia, Patos de Minas, MG, Brazil; 6 Department of Nutrition and Dietetics, Faculty of Medicine and Health Sciences, Universiti Putra Malaysia, 43400 Serdang, Selangor, Malaysia; 7 Laboratory of Medical Biochemistry, Faculty of Medicine, University of Annaba, Algeria

**Keywords:** gene surgery, CRISPR-Cas, cancer, diabetes, obesity

## Abstract

Gene therapy became in last decade a new emerging therapeutic era showing promising results against different diseases such as cancer, cardiovascular diseases, diabetes, and neurological disorders. Recently, the genome editing technique for eukaryotic cells called CRISPR-Cas (Clustered Regulatory Interspaced Short Palindromic Repeats) has enriched the field of gene surgery with enhanced applications. In the present review, we summarized the different applications of gene surgery for treating human diseases such as cancer, diabetes, nervous, and cardiovascular diseases, besides the molecular mechanisms involved in these important effects. Several studies support the important therapeutic applications of gene surgery in a large number of health disorders and diseases including β-thalassemia, cancer, immunodeficiencies, diabetes, and neurological disorders. In diabetes, gene surgery was shown to be effective in type 1 diabetes by triggering different signaling pathways. Furthermore, gene surgery, especially that using CRISPR-Cas possessed important application on diagnosis, screening and treatment of several cancers such as lung, liver, pancreatic and colorectal cancer. Nevertheless, gene surgery still presents some limitations such as the design difficulties and costs regarding ZFNs (Zinc Finger Nucleases) and TALENs (Transcription Activator-Like Effector Nucleases) use, off-target effects, low transfection efficiency, *in vivo* delivery-safety and ethical issues.

## Introduction

A gene is an expressed DNA sequence that can code for a protein or generate non-coding RNA sequences (ncRNA). In general, eukaryotic genes consist of an alternation of multiple exons (coding sequence, transcript and translated) and introns, besides the regulation and promoter sequences. Although introns are considered noncoding or intervening sequences, it has been demonstrated that they are able to regulate gene expression through the intron-mediated enhancement characterized by an mRNA accumulation (Shaul, 2017[159]; Gallegos and Rose, 2015[62]).

Gene's mutations are considered causes of several diseases such as cancer. Indeed, for example, DNA methylation has been demonstrated to be involved in at least 18 cancer types (Chen et al., 2017[30]). Genomic instability including chromosome and microsatellite instabilities and increased frequencies of base-pair mutation is a hallmark of most cancer cells (Yao and Dai, 2014[186]). Recently, in the most comprehensive study of gene mutations, 3400 driver mutations were found to be associated with 33 various cancer types (Bailey et al., 2018[9]). 

**Breast cancer** has been linked to several mutations of the BRCA genes. BRCA 1 and BRCA 2, considered as tumor suppressor genes, are involved in the maintaining of genetic stability by repairing DNA damages caused by environmental agents or even chromosomal events. BRCA 1 and 2 repair double-strand DNA breaks by homologous recombination repair. BRCA1 participates in DNA repair by neutralizing the Non-homologous end joining (NHEJ) factor 53BP1, whereas BRCA2 is involved in RAD51 filament assembly onto ssDNA (Chen et al., 2018[28]). Consequently, BRCA mutations resulting in higher genetic instability and gross chromosomal rearrangements, are linked to an enhanced risk of several malignancies such as breast, ovarian, prostate and/pancreatic cancers (Solinas et al., 2019[162]; Foulkes et al., 2016[58]). BRCA mutations cause homologous recombination repair deficiency leading to carcinogenesis (Liposits et al., 2019[117]). BRCA1/2 mutations are responsible for 15 % of triple-negative breast cancer cases and of 50 % of breast cancer cases in individuals with a strong family history (den Brok et al., 2017[46]). Besides their involvement in genetic predisposition to cancers, BRCA 1/2 mutations are also considered promising targets of homologous recombination directed therapies such as PARP inhibitors (olaparib, rucaparib, and talazoparib for both breast and ovarian cancers) via the synthetic lethality (Sporikova et al., 2018[163]; Riaz et al., 2017[147]).

**Diabetes mellitus** is a polygenic disease involving upregulation and/or downregulation of a genes complex network (ADAMTS9, CDC123/CAMKID, JAZF1, NOTCH2, THADA, TSPAN8/LGRS, PPARG, ABCC8 and KCNJ11, PPARG, HMGA1, HNF4A, IRS1, HNF1A, AKT2, TCF7L2, IGF2BP2, MAPK8IP1, IRS2, NEUROD1, HNF1B, UBC, GCK, FGFR3, others) responsible for the occurrence, progression and complications of the disease (Gupta and Vadde, 2019[73]; Peravali et al., 2019[140]). 

Type 1 diabetes mellitus is an auto-immune disease characterized by failure in insulin production as a result of pancreatic insulin-secreting islet β cells destruction. In spite of its complex etiology, it has been demonstrated that various genes are involved in the apparition and development of this chronic disease. Indeed, downregulation of f insulin-like growth factor 1 (IGF1) and glucose-6-phosphatase (G6Pase) genes, and also upregulation of the regenerating islet-derived protein 3 gamma gene (Reg3g) have been associated with type 1 diabetes mellitus (Chellappan et al., 2018[27]).

On the other hand, several genes and mutations on they were found to play a crucial role in the development of type 2 diabetes. Indeed, it has been found that among 157 genes studied in type 2 diabetic patients, 124 had at least one mutation (Al-Rubeaan et al., 2018[4]). Among gene deregulations associated with type 2 diabetes are SIRT1, transcription factor 7-like2 (TCF7L2), Potassium channel gene (KCNJ11), Glucocorticoid receptor (GRL), and Norepinephrine transporter (NET) (Karbasforooshan and Karimi, 2017[91]).

The high mobility group AT-hook1 (HMGA1) gene codes for a 10 kDa multifunctional non-histone chromatin protein implicated in various biological processes such as apoptosis, cell differentiation and DNA repair (Zhang et al., 2018[193]; Fujikane et al., 2016[61]). In addition to its role in oncology and inflammation, HMGA1 was found to be involved in the transcription of insulin receptor INSR (as a sensor and regulator of insulin signaling), glucose homeostasis and therefore in type 2 diabetes development. It has been reported that HMGA1 deficiency leads to insulin resistance and type 2 diabetes both in humans and mice (Chiefari et al., 2018[32]; Xue et al., 2018[184]).

Several **nervous disorders** have been found to be associated with gene mutations and/or deregulations (Wang et al., 2019[173]; Eid et al., 2019[54]). Parkinson's disease characterized by dopamine deficiency has been linked to mutations in 10 genes: SNCA, LRRK2, PRKN, PINK1, DJ-1, ATP13A2, PLA2G6, FBXO7, GIGYF2, and UCHL1. Furthermore, genetic susceptibility to the disease has been shown to be related to various genes such as NR4A2, SNCAIP, APOE, MAPT, and GBA (Selvaraj and Piramanayagam, 2019[156]). On the other hand, genetic mutations have been linked to Alzheimer disease. It has been reported that at least, 117 genes are related to Alzheimer disease (Grimm et al., 2019[71]). Indeed, autosomal-dominant early-onset Alzheimer disease has been found to be attributed to three genetic mutations: amyloid protein precursor (APP), presenilin-1 (PSEN1), and presenilin-2 (PSEN2) (Lanoiselée et al., 2017[104]). Besides, apolipoprotein E (ApoE) isomers have been demonstrated to be related to the development of the disease in different populations (Ramadan et al., 2019[146]). Sporadic Alzheimer's disease susceptibility has been found to be attributed to several genes such as BIN1, CLU, PICALM, ABCA7, ABCG1, and SORL1 (Picard et al., 2018[142]). 

**Gene therapy** refers to the insertion of normal genetic material into the cells of the patient to induce the expression of a genetic sequence responsible to achieve a therapeutic effect. Gene augmentation therapy (gene replacement or addition), genes specific targeted therapy, and genome editing or correction therapy are possible approaches as gene therapy (Lee et al., 2018[107]; Lin et al., 2018[113]). 

With more than 2597 clinical trials carried out in 38 countries (Ginn et al., 2018[67]), gene therapy continues to provide promising positive results regarding the treatment of various diseases such as retinal diseases, primary immunodeficiencies, neurological disorders, β-thalassemia, hemophilia, diabetes, and cancers (Tan et al., 2019[167]; Kumar et al., 2016[100]).

## Gene Surgery

The genome-editing technique for eukaryotic cells called CRISPR-Cas has advanced the field of genetic engineering owing to the potential to generate cellular models more related to *in vivo* system than ever before (Dai et al., 2018[43]; Erard et al., 2017[55]; Wangensteen et al., 2018[174]; Long et al., 2014[121]; Wu et al., 2013[181]; Freiermuth et al., 2018[59]). Described in bacteria and archaea (Ishino et al., 1987[80]; Mojica et al., 2000[130]), this system possesses a particular configuration of a Clustered Regulatory Interspaced Short Palindromic Repeats (CRISPR) and a endonuclease regulatory protein called as Cas (CRISPR-associated) (Jansen et al., 2002[82]; Semenova et al., 2011[157]; Hsu et al., 2014[78]). CRISPR locus was first described as short repeats interspaced by unique extrachromosomal sequences from host organisms that confer immunity against plasmid or bacteriophage infection (Bolotin et al., 2005[16]). The spacers match with sequences from phages and plasmids (Wei et al. 2013[175]) rising the hypothesis that the CRISPR-Cas mechanism is an adaptive immune response of prokaryotes (Barrangou et al., 2007[12]; Sampson and Weiss, 2014[151]; Sürün et al., 2018[166]). 

Briefly, the CRISPR-Cas defense is based on adaptation, biogenesis of crRNA (CRISPR-derived RNA) and action against the invader. In the first phase, a new spacer is acquired in CRISPR locus through cleavage and integration of a foreign DNA. In the second step, the CRISPR is transcribed in a long pre-crRNA which is processed in many small crRNAs, each one with a distinct spacer flanked by repeated fragments. Finally, Cas proteins interact with crRNAs, that drive the cleavages of invader DNA by the endonuclease (Wiedenheft et al., 2012[177]; Karvelis et al., 2013[92]; Sampson and Weiss, 2014[151]; Wiles et al., 2015[178]; Sürün et al., 2018[166]).

Multiple and diverse CRISPR-Cas systems are defined by different Cas proteins and crRNA biogenesis (Makarova et al., 2015[125]; Carte et al., 2014[23]). In Type I, Cas6 cleaves the pre-crRNA and the mature guide combines with a CRISPR-associated complex for antiviral defense (Cascade). The sequence-specific cleavage is mediated by the Cas3 protein which needs a short DNA motif called *Protospacer Adjunct Motif* (PAM) for target recognition. In Type III the pre-crRNA is processed by Cas6, however, the ribonucleoprotein complex includes Cas10 and the accessory genes *Csm *(III-A - DNA cleavage) and *Crm* (III-B - RNA cleavage) (Makarova et al., 2015[125]; Karvelis et al., 2013[92]; Jiang and Marraffini, 2015[85]; Carte et al., 2014[23]). Type II is the simplest mechanism that consists of a crRNA maturation pathway including an additional *Trans-activating CRISPR RNA molecule* (tracrRNA) and the host RNase III. Besides, a single Cas9 provides target DNA cleavage (Karvelis et al., 2013[92]; Cong et al., 2013[39]; Carte et al., 2014[23]). 

In this system, CRISPR locus produces the tracrRNA with repeat sequences annealing to the repeat sequences of pre-crRNA. The Cas9 binds the tracrRNA and the dsRNA (tracrRNA plus pre-crRNA) is cleavage by RNase III, generating a Cas9 loaded with tracrRNA and crRNA guide (Jiang and Marraffini, 2015[85]; Carte et al., 2014[23]; Jinek et al., 2012[87]). The endonuclease Cas9 needs the PAM motif in the 3'end of the target (Jinek et al., 2012[87]; La Russa and Qi, 2015[103]; Ehrke-Schulz et al., 2017[53]). The most efficient PAM sequence essential for Cas9 binding to the DNA is any nucleotide together with two guanines (NGG) (Jinek et al., 2012[87]; La Russa and Qi, 2015[103]; Anders et al., 2014[5]; Larson et al., 2013[105]). The tracrRNA/crRNA/ Cas9 ribonucleoprotein scans the target genome for PAM sequences and binds immediately upstream of this motif (Anders et al., 2014[5]; Yuen et al., 2017[190]). Cas9 has two endonuclease domains (RuvC and HNH) that cut both DNA strands and tracrRNA acts as a cofactor (Jiang and Marraffini, 2015[85]; Jinek et al., 2012[87]). The RuvC, HNH, and PAM-interacting domains are in the nuclease (NUC) lobe of Cas9 that contains also the recognition lobe (REC) (Dai et al., 2018[43]; Jinek et al., 2012[87]).

Based on this natural mechanism, CRISPR technique was developed involving a nuclease (usually Cas9 from *Streptococcus pyogenes*), a single-guide RNA (sgRNA) and the target in the genome (Gilbert et al., 2013[66]; Hsu et al., 2014[78]; Platt et al., 2014[143]; Pelletier et al., 2015[139]; Kocher et al., 2017[96]; Sürün et al., 2018[166]). The sgRNA is a chimeric molecule made up of crRNA and tracrRNA, which is engineered and programmed to target with the interested sequence, responsible for the duplex with the target of DNA, by Watson and Crick base pairs (Hsu et al., 2014[78]; Jinek et al., 2012[87]; La Russa and Qi, 2015[103]; Perez-Pinera et al., 2013[141]). The sgRNA contains a seed-sequence with about eight nucleotides in the 5' end, and the 3'end forms a scaffold with approximately 80 nucleotides in which Cas9 can fold into an active form (Semenova et al., 2011[157]; Zeng et al., 2018[191]; Jinek et al., 2012[87]). Stable RNA: DNA hybrid (sgRNA plus target DNA) leads to R-loop formation and conformational change of the enzyme (Zeng et al., 2018[191]; Yuen et al., 2017[190]; Hsu et al., 2014[78]). Finally, coordinated firing of both RuvC and HNH nuclease domains results in DNA cleavage (Jiang and Marraffini, 2015[85]). 

After site recognition through sgRNA, the Cas9 cleaves the target generating blunt-ended double-strand breaks (DSB) (Markossian et al., 2018[127]; Billon et al., 2017[14]; Jinek et al., 2012[87]; La Russa and Qi, 2015[103]; Komor et al., 2017[97]; Jubair and McMillan, 2017[89]; Pelletier et al., 2015[139]). The DSB can be repaired by error-prone NHEJ or by the high-fidelity homology-directed repair (HDR) (Hsu et al., 2014[78]; Pelletier et al., 2015[139]; Kocher et al., 2017[96]). The first pathway may generate gene knockout and may be expanded to multiple target sites mediating larger editing in the genomes. HDR, through recombination, may create allelic substitution, with a promised perspective in disease therapy (Jubair and McMillan, 2017[89]; Kocher et al., 2017[96]; Rivera-Torres et al., 2017[148]).

Variants of wild Cas9 have been developed. Inactivation of one of the catalytic domain leads to Cas9 nickase (Cas9n) strategy which cleaves one of the two strands of target DNA (Gasiunas et al., 2012[64]; Nakajima et al., 2018[134]; Zhao et al., 2017[194]; Kurihara et al., 2017[101]; Sakuma et al., 2016[150]). The catalytically dead version of Cas9 (dCas9) has both nuclease domains mutated and has been employed for transcription and epigenetic regulation (Gilbert et al., 2013[66]; Pulecio et al., 2017[145]; Chen et al., 2017[30]; Hao et al., 2017[75]; Adamson et al., 2016[2]; Klann et al., 2017[93]). dCas9 may bind to promoter sequences or open reading frames modulating gene expression in DNA level (Lundh et al., 2017[122]; Gilbert et al., 2014[65]; Cheng et al., 2013[31]; Maeder et al., 2013[124]; Mandegar et al., 2016[126]; Duellman et al., 2017[51]). This variation of CRISPR-Cas technique can act in ncRNAs as microRNAs (miRNAs) and any other genomic region and enables fusing of fluorescent proteins for DNA visualization (Jiang and Marraffini, 2015[85]; Liu et al., 2017[118]; Ye et al., 2017[187]; Knight et al., 2018[94]; Dominguez et al., 2016[47]). The understanding of conformational R-loop dynamics and RNA: DNA interactions are necessary for a better design of specific sgRNAs and their variants Cas9/dCas9-based (Josephs et al., 2015[88]).

The molecular genomic strategy CRISPR-Cas9-based has been exploited for biological and medical applications (Komor et al., 2017[97]; Kwarteng et al., 2017[102]; Kurihara et al., 2017[101]; Sakuma et al., 2016[150]; Zhao et al., 2017[194]; Josephs et al., 2015[88]). Optimization of 5' sequence of the sgRNA and chemical modifications of Cas9 have been achieved to control off-targets and unspecific cleavages, maximizing the on-target activity (Chira et al., 2017[34]; Cao et al., 2016[19]; Josephs et al., 2015[88]). Moreover, Cas9 coding sequence has been introduced in different viral vector including retroviral, lentiviral, and adeno-associated (Senís et al., 2014[158]; Sürün et al., 2018[166]), as well as in high-capacity adenoviral vectors in order to improve editing machine (Ehrke-Schulz et al., 2017[53]; Hsu et al., 2014[78]). 

In fact, CRISPR-Cas9 has overcoming other nuclease-based systems, such as Zinc Finger Nucleases (ZFNs) and Transcription Activator-Like Effector Nucleases (TALENs) (Kwarteng et al., 2017[102]; Chira et al., 2017[34]; Hsu et al., 2014[78]; La Russa and Qi, 2015[103]; Larson et al., 2013[105]; Pelletier et al., 2015[139]). It has been explored as a molecular tool for basic and applied research with therapy purposes in human diseases and life sciences (La Russa and Qi, 2015[103]; Larson et al., 2013[105]; Pelletier et al., 2015[139]; Aquino-Jarquin, 2017[6]). Nanotechnology has been reached in CRISPR-Cas9 to increase edited cells (Carlson-Stevermer et al., 2017[21]). This technique represents an expanding area for interrogating genetic elements and functions, controlling site-directed mutation and modulating gene expression (Jubair and McMillan, 2017[89]).

### Applications of gene surgery

#### Genome editing

The DNA recombinant technology developed in the 1970s remarkable the new biology, enabling to engineer DNA molecules. This modern biotechnology established linkages between genetic variations and biological phenotypes (Ishino et al., 1987[80]; Mojica et al., 2000[130]; Hsu et al., 2014[78]). The traditional gene therapy based on vector-mediated overexpression methods was developed to supply the cells with the functional gene copies for monogenic recessive diseases (Hsu et al., 2014[78]). Currently, the gene therapy using the CRISPR-Cas9 system is suited for recessive and dominant inherited monogenic disorders (Jiang and Marraffini, 2015[85]; Kocher et al., 2017[96]; Sürün et al., 2018[166]). 

Virtually, CRISPR-Cas9 can repair endogenous mutations and insert specific or random mutations (Pelletier et al., 2015[139]). In this sense, Cas9 may be used to correct single nucleotide polymorphism (SNP) where at least one copy of the functional gene can be expressed in its natural environment (Hsu et al., 2014[78]). If the monogenic disease is due to the duplication of genomic sequences, Cas9 may be exploited to delete the duplicated sequences. For the expansion of trinucleotide repeats two simultaneous DSBs may excise the repeat region (Hsu et al., 2014[78]). For monogenic dominant disorders, the NHEJ may inactivate the mutant allele, and the sgRNA will be designed targeting to the specific allele with the SNP (Hsu et al., 2014[78]). Rare diseases can also benefit from Cas9 technology (Pelletier et al., 2015[139]; Kocher et al., 2017[96]). Using this system is possible to prevent non-genomic elements as virus (Kwarteng et al., 2017[102]; Sakuma et al., 2016[150]; Pelletier et al., 2015[139]), by the promotion of mutation and repair NHEJ-mediated, circumventing infection without the need for continuing treatment (Kwarteng et al., 2017[102]; Pelletier et al., 2015[139]). The CRISPR-Cas9 multiplexed with the Cas9n vector can reduce viral replication (Sakuma et al., 2016[150]). 

The perturbation of multiple genes simultaneously can also allow the discovery of new generation drugs and therapeutics, tested in the cell or transgenic animal created through CRISPR-Cas pathway (Jiang and Marraffini, 2015[85], Hsu et al., 2014[78], Pelletier et al., 2015[139], Kocher et al., 2017[96]). The animal models CRISPR-Cas9-based have been produced to overcome the delivery challenges and to provide tools for *in vivo* or *ex vivo* study of mutations associated with human diseases (Dai et al., 2018[43]; Erard et al., 2017[55]; Jiang and Marraffini, 2015[85]; Long et al., 2014[121]; Wangensteen et al., 2018[174]; Wu et al., 2013[181]; Markossian et al., 2018[127]; Hsu et al., 2014[78]; Platt et al., 2014[143]; Kocher et al., 2017[96]; Shi et al., 2017[160]). The most well-known objective considering CRISPR-Cas9 involves the generation of frameshift indels (insertion or deletion) of random mutations in single or multiple genes at once to better understand cellular physiology (Komor et al., 2017[97]; Markossian et al., 2018[127]; Billon et al., 2017[14]; Pelletier et al., 2015[139]; Jiang and Marraffini, 2015[85]). The development of a multi-color and multi-locus Cas9 proteins or sgRNAs by labeling with fluorescent molecules can be an alternative method to the traditional probe DNA labeling, providing living cell imaging about complex chromosomal architecture and nuclear organization (Jiang and Marraffini, 2015[85]; Liu et al., 2017[118]; Ye et al., 2017[187]; Dominguez et al., 2016[47]; Knight et al., 2018[94]; Hsu et al., 2014[78]).

Genome editing can promote either chromosomal biallelic or monoallelic frameshift mutations (Platt et al., 2014[143]). Chromosomal alterations are efficiently generated by large insertions or deletions that disrupt intra or intergenic regions by DSBs (Zuo et al., 2017[197]; Pelletier et al., 2015[139]). Moreover, chromosomal translocation can also be produced using an HDR oligonucleotide with sequence homology to both chromosomal locus and co-injected with CRISPR-Cas9 (Pelletier et al., 2015[139]).

#### Genetic and epigenetic regulation of gene expression

In the central dogma of molecular biology is the combined control of gene expression coordinating the events of gene transcription and translation, as the turnover of RNAs and proteins. The dysregulation of gene expression often results in cancer, metabolic disorders, cardiovascular and other human diseases (Komor et al., 2017[97]; La Russa and Qi, 2015[103]; Chen and Qi, 2017[29]). Significant efforts have been devoted to engineering transcriptional factors to control biological processes and, thereby, create new therapeutic strategies (Gilbert et al., 2013[66]). 

The regulatory strategies using dCas9 can produce levels of gene control without precedent (La Russa and Qi, 2015[103]). The control of the transcriptional regulation is crucial for understanding cellular behavior and dCas9 targeting regions upstream or downstream of the transcription start site modulates cellular response (Braun et al., 2016[17]). Regulation of gene expression by CRISPR-dCas9 system is termed as CRISPRa for gene activation or CRISPRi for gene repression (Komor et al., 2017[97]; Gilbert et al., 2013[66]; La Russa and Qi, 2015[103]; Perez-Pinera et al., 2013[141]; Chen and Qi, 2017[29]), becoming versatile when multiple sgRNAs/dCas9 fusions are multiplexed to regulate multiple targets within the same pathway (La Russa and Qi, 2015[103]). For example, dCas9 may be employed as a transporter protein directed to specific or multiple loci guided by sgRNA, activating or repressing gene expression (CRISPRa/i) (Komor et al., 2017[97]; Chen and Qi, 2017[29]; La Russa and Qi, 2015[103]; Perez-Pinera et al., 2013[141]).

However, it is noteworthy that genome organization in eukaryotes is complex and DNA regulatory elements can be far from the transcriptional start point. Epigenome also coordinates gene expression, and chromatin remodeling and chemical modifications of histones must be taken into account for expression regulation (Gilbert et al., 2013[66]). Three-dimensional organization of chromatins exerts a putative role in gene expression, and how chromatin loops are involved in this process needs to be clarified. In this field, CRISPR/dCas9 may interfere in nuclear architecture demonstrating the power of this system to reorganize chromatin loops (Morgan et al., 2017[131]). 

The gene expression by epigenome editing can be regulated by dCas9:sgRNA complex using combinations of synthetic transcription factors to bind near the transcriptional endogenous human gene promoters with a relevant role in medicine (Komor et al., 2017[97]; Cheng et al., 2013[31]; Perez-Pinera et al., 2013[141]; Polstein and Gersbach, 2015[144]). The co-recruitment of multiple activators may be the strategies to produce the second generation of CRISPRa (La Russa and Qi, 2015[103]). The transcriptional activation domain VP64 from Herpes Simplex Viral Protein 16, fused with dCas9 (dCas9-VP64) was used to modulate the expression of a wide gene (Komor et al., 2017[97]; Guo et al., 2017[72]; Balboa et al., 2015[10]; Perez-Pinera et al., 2013[141]). The dCas9 fused to a histone acetyltransferase stabilized the expression of a transcription factor regulated by epigenetic modification in inflammatory conditions modulating cellular response (Okada et al., 2017[136]).

Alternatively, CRISPRi is an approach for perturbing gene expression with selectivity to RNA polymerase, transcription elongation and/or with the sgRNA binding to specific DNA targeting the protein-coding region on a genome-wide scale (Jiang and Marraffini, 2015[85]; Chen and Qi, 2017[29]). The use of CRISPRi represents an exciting field to the counterpart RNA interference (RNAi) (Jiang and Marraffini, 2015[85]; La Russa and Qi, 2015[103]; Larson et al., 2013[105]). The use of quantitative fluorescence assays and native elongating transcript sequencing was described to produce a protocol for gene silencing at the transcriptional level through sgRNA providing the repression activity of CRISPRi as a complementary approach to RNAi (Larson et al., 2013[105]).

The advantage and disadvantages of the use of RNAi and CRISPRi need to be systematically evaluated about the effectivity in each different type of cell. In the CRISPRi system, the bioinformatics modeling identifying high-confidence hits to generate sgRNAs directed to the target and testing large pools is important to find the ideal sgRNAs reducing off-targets (Erard et al., 2017[55]; La Russa and Qi, 2015[103]). The success of the CRISPR-Cas/dCas system has empowering researchers to better understand prokaryotic and eukaryotic genome functions and biology system (Jiang and Marraffini, 2015[85]; Hsu et al., 2014[78]). 

### Molecular mechanism-mediated gene surgery

#### Obesity

Obesity affects nearly 500 million people worldwide and is at risk of developing cardiovascular disorders, cancer, and type 2 diabetes (Garcίa-Jiménez et al., 2016[63]). Obesity is primarily due to a positive energy balance, whereby the energy consumption exceeds expenditure, leading to energy storage, predominantly as lipids in white adipocytes (Claussnitzer et al., 2015[37]). Notably, severely obese patients had medical costs that were $1980 higher annually per capita, compared to that of individuals within a normal body mass index (BMI) (Julie and Jacob, 2015[90]). BMI comprised of strong genetic components (40 to 80 % heritability) with several genes expressed in the hypothalamus and plays a crucial role in appetite regulation (Locke et al., 2015[120]).

Investigators at MIT, Havard University, and the Broad Institute of Cambridge had identified a variant of the fat mass and obesity-associated (*FTO*) gene that is responsible for the development of fat cells in the body and thus it is considered as one of the primary factors for weight gain (Claussnitzer et al., 2015[37]). However, what is worth to mention here was not the isolation of the *FTO* gene variant, the researchers were able to modify the living cells in mice that carrying variant using CRISPR-Cas9 (Maclean, 2016[123]). 

Using DNA editing tools, they have identified a genetic switch that facilitates the regulation of body metabolism. The switch governs whether common fat cells burn energy or store it as fat (Vogel, 2015[170]). CRISPR/Cas9 also can be used to convert the obesity-promoting *FTO* gene in adipocyte precursor cells. The treated cells had lowered *IRX3 *and *IRX5* expression, and ultimately spun up the energy-burning machinery (Claussnitzer et al., 2015[37]).

In another study, Yue et al. (2017[189]) reported that CRISPR-mediated genome editing controlled the release of glucagon-like peptide 1 (GLP1), a crucial incretin that regulates blood glucose homeostasis. In this study, GLP-1 induction from engineered mouse cells grafted onto immunocompetent hosts would be triggered by the administration of the antibiotic doxycycline, which subsequently reduces the appetite and decreases the high fat diet-induced obesity. Importantly, GLP-1 induction can reverse the high-fat diet-induced weight gain. Therefore, the ability in the manipulation of the uncovered pathway, such as overexpression or knockdown of the upstream regulator *ARID5B*, a genome editing of the predicted causal variant rs1421085, and overexpression or knockdown of target genes *IRX5 *and* IRX3*, had a prominent effect on obesity phenotypes.

#### Cancer

As a consequence of the global rise of obesity, the current and future burden of cancers associated with obesity tends to increase. This is not only affecting the occurrence of cancer, a high prevalence of obesity also affects prognosis among cancer survivors (Arnold et al., 2016[7]). Cancer is a disease featured by multiple epigenetic and genetic alterations in tumor suppressor genes and oncogenes (Deben et al., 2016[45]). All cancers exert stepwise mutations that allow the cells to multiply and exhibit their malignancy features (Economides et al., 2017[52]; Xu et al., 2017[182]). Cancer is genetically complex with hundreds of translocations, mutations, and chromosome losses and gains per tumor (Greer et al., 2017[70]). Inappropriate activation of Wnt pathway has linked with different types of cancers. Loss of the adenomatous polyposis coli (*APC*) gene function induces a premalignant precursor lesion, which is a hallmark of human colorectal cancers (CRC) (Novellasdemunt et al., 2015[135]). Given the advance in comprehensive structural characterization in cancer genomes and sequencing technology (Vogelstein et al., 2013[171]), the mutations in the Wnt pathway frequently implicated in human cancers (Duchartre et al., 2016[50]). Although the fact that most of the pathway components have been characterized, the function of Wnt pathway within the context of cancer biology is intriguingly complex and yet fully understood (Zhan et al., 2017[192]). 

Over the past decades, research and development on genome engineering technologies have made it possible to modify or precisely delete specific DNA sequences in the genome of the cells or animal models (Sánchez-Rivera and Jacks, 2015[152]). The ability to correct such mutations is a primary goal in cancer treatment. There is substantive evidence based on the role of genome editing with CRISPR/Cas9 as a rapid genetic manipulation approach in any genomic locus (Dow et al., 2015[49]).

It is also demonstrated that CRISPR/ Cas9 system has been used to generate cancer cells from mouse primary cells or *in vivo *tissue (Heckl et al., 2014[76]; Xue et al., 2014[183]). Even though the requirement of Wnt-β-catenin signaling axis in RNF43-mutated pancreatic ductal adenocarcinoma (PDAC) (Jiang et al., 2013[86]), the genetic complexity in this pathway for example co-receptors and intracellular signaling components, frizzled (FZD) receptors and Wnt ligands made the prediction of this therapeutic targets become difficult. Therefore, identification of this specific genetic vulnerability using genome-wide CRISPR-based genetic screens may provide an unbiased and powerful means to identify context-specific fitness genes that can be harnessed in the treatment of cancer. Genome screening using CRISPR/Cas9 enables the detection of the Wnt-FZD5 signaling circuit in an RNF43-mutant pancreatic tumor (Steinhart et al., 2017[164]).

Treating de novo Kaposi's sarcoma-associated herpesvirus (KSHV)-infected endothelial cells with epilipoxinand lipoxinexhibits an anti-inflammatory effect by reducing the nuclear factor kappa B(NF-κB), extracellular signal-regulated kinase ½ (ERK1/2), cyclooxygenase-2(COX-2), 5-lipoxygenase, and Akt expression. Furthermore, treatment with lipoxinusing CRISPR/ Cas9 technology-mediated lipoxin A4 receptor/formylpeptidyl receptor (ALX/FPR) gene deletion implied the efficiency of lipoxin receptor ALX in lipoxin signaling (Chandrasekharan et al., 2016[26]). In this study, a viral miRNA cluster has been identified as an important factor in the downregulation of lipoxin A4 secretion in the host cells (Chandrasekharan et al., 2016[26]). Additionally, De Ravin et al. (2017[44]) also demonstrated that CRISPR/Cas9 could repair a mutation in the CYBB gene of CD34+ hematopoietic stem and progenitor cells (HSPCs) for those of the patients with immunodeficiency disorder X-linked chronic granulomatous disease (X-CGD). The beta-hemoglobinopathies, such as beta-thalassemia and sickle cell disease are caused by beta-globin (HBB) gene mutations which had affected millions of people worldwide. Surprisingly, using CRISPR-based methodology the *ex vivo* gene can be corrected in patient-originated hematopoietic stem cells after autologous transplantation. 

The miRNAs play a crucial role in the development of breast cancer. An earlier study has reported that miRNAs expression was changed significantly in cancer cell lines and tumor tissues (Ji et al., 2017[83]). miRNA dysfunction is a biomarker in various pathological diseases such as cancer (Ji et al., 2017[83]). Therefore, miRNA has been recognized as a robust biomarker in ovarian, prostate, and ovarian cancers for therapeutic targeting (Smith et al., 2017[161]). This potential therapeutic effect has been demonstrated by Abdollah et al. (2017[1]), who found that the expression of miR-130a-5p was downregulated in breast cancer (MCF7) cell line using CRISPR silencing system. It was also revealed from several *in vitro *and *in vivo *studies that this editing tool has potential in inhibition of cancer cell growth and increases cell apoptosis (Liu et al., 2014[119]; Zhen et al., 2017[195]). Importantly, CRISPR systems are being applied to ameliorate genetic disorders in animals and seem to be employed in human diseases (Barrangou and Doudna, 2016[11]). The prospects of this gene-editing technology trigger a biomedical duel in China when the CRISPR-Cas9 technique was first introduced to a patient with aggressive lung cancer at West China Hospital, Chengdu (Cyranoski, 2016[42]).

In addition to the significant therapeutic approach of in vivo somatic genome editing to alter genetic defects (Long et al., 2014[121]; Yin et al., 2014[188]), its applications in cancer modeling have been convincingly reported (Table 1(Tab. 1); References in Table 1: Aubrey et al., 2015[8]; Blasco et al., 2014[15]; Chiou et al., 2015[33]; Dow et al., 2015[49]; Xue et al., 2014[183]; Zuckermann et al., 2015[196]). For instance, several groups have demonstrated that direct in vivo delivery of the relevant gRNA and Cas9 to the specific tissue using viral vectors or naked DNA can be used to initiate tumorigenesis and suppress somatically tumor-suppressor genes (Platt et al., 2014[143]; Xue et al., 2014[183]; Chiou et al., 2015[33]; Zuckermann et al., 2015[196]). This approach has been successfully utilized to inactivate the tumor suppressor genes such as liver kinase B1 (Lkb1), transformation-related protein 53 (Tp53), phosphatase and tensin homolog (Pten), and adenomatous polyposis coli (APC) in the lungs of adult mice (Platt et al., 2014[143]). Preclinical findings from *in vitro *and *in vivo *studies on mechanisms involved in cancer suggest that the CRISPR-Cas9 technique could modulate pathway involving in cancers. Targeting the cancer cells nucleotide sequences with the cellular genome is an attractive approach. Collectively, the ability to correct the cancer-associated mutations has been identified as a powerful treatment option. These techniques facilitate in the correction of mutations in the cancer cells.

#### Diabetes

Diabetes is a chronic and metabolic disease featured by elevation of blood glucose levels, which cause serious damage to the blood vessels, eyes, heart, nerves, and kidneys (World Health Organization, 2017[179]). According to the World Health Organization (2017[179]), there are 422 million adults having diabetes with 1.6 million deaths annually (World Health Organization, 2017[179]). CRISPR/Cas9 could alter the gene that is responsible for encoding a hormone known as glucagon-like peptide-1 (GLP-1). GLP-1 promotes the release of insulin and facilitates in the reduction of glucose from the blood (Yue et al., 2017[189]). This therapeutic potential was not only exhibited in **in vivo** genome editing, its applications in Type 1 diabetes in a mouse modeling have also been demonstrated. For example, *Ptpn22^R619W^* mice showed an increase in insulin autoantibodies and high susceptibility of Type 1 diabetes (Lin et al., 2016[114]). Notably, inhibition of FOS/JUN pathway either through CRISPR-mediated suppression of FOS rescued the inability of *CDKAL1* null cells to alter glycemia in streptozotocin-diabetic mice (Rutter, 2016[149]). Taken together, these editing techniques hold great promise in future use in the treatment of diabetes. 

#### Cardiovascular diseases

Cardiovascular diseases (CVDs) are a major health problem and the leading cause of death globally (Go et al., 2013[68]). Several polymorphisms have already been identified and implicated in the pathogenesis of CVDs and more continue to be located by scientists every day (Fiatal and Ádány, 2018[56]). The emergence of gene-surgery technologies has given researchers new approaches to study and treat the common complex CVDs such as inherited cardiomyopathies, valvular diseases, primary arrhythmogenic conditions, congenital heart syndromes, hypercholesterolemia and atherosclerotic heart disease, hypertensive syndromes, and heart failure with preserved/reduced ejection fraction (Pasipoularides, 2018[137]).

The three universally used tools are Zinc Finger Nucleases (ZFNs), Transcription Activator-Like Effector Nucleases (TALENs), and the Clustered Regularly Interspaced Short Palindromic Repeats (CRISPR)-associated Cas9 (CRISPR/Cas9) system (Termglinchan et al., 2016[168]). Each system has its own advantages and limitations and has been applied in various scientific fields to increase the understanding of normal gene regulation and the pathophysiology of the disease, as well as to facilitate the development of novel therapies (Seeger et al., 2017[154]).

One of the major goals of gene surgery is replacing defective, down-regulated, or missing genes with normal, enabling the normal function or, alternatively, adds a new gene to restore or improve well-being. In fact, there are different approaches for using gene editing according to two categories of CVDs: diseases caused by primary cardiomyopathy that could be corrected by genome editing within the cardiac muscle, and diseases secondarily caused by extracardiac influences (Strong and Musunuru, 2017[165]). In the first category, the most likely example are the inherited cardiomyopathies, such as familial hypertrophic cardiomyopathy and dilated cardiomyopathy. In a large proportion of patients, these cardiomyopathies are caused by single dominant mutations within sarcomere genes (Musunuru, 2017[133]). In principle, these diseases could be prevented or arrested by two different strategies: correction of the mutant allele by homology-directed repair (HDR), or allele-specific gene disruption via Non-Homologous End-Joining (NHEJ) strategy, removing the dominant influence of the mutant allele (Jiang et al., 2013[84][86]). 

The first strategy might be extremely challenging to achieve owing to limited HDR in adult cardiomyocytes. An approach similar to the second strategy has been achieved in a mouse model through the use of RNA interference, suggesting the viability of the NHEJ genome-editing approach (Coelho et al., 2013[38]). The success of the NHEJ approach would depend on being able to achieve stringent specificity for the mutant allele, which usually differs from the wild-type allele by only a single base pair. For CRISPR/Cas9, this specificity would be most feasible if the causal mutation created a point accepted mutation (PAM) site that was not present in the wild-type allele (Chong et al., 2014[35]). 

The second category of diseases includes the treatment of hypercholesterolemia to reduce the risk of myocardial infarction, via the disruption of proprotein convertase subtilisin/kexin type 9 (*PCSK9) *or other lipoprotein regulators in the liver (Cai et al., 2016[18]). When PCSK9 is dysregulated, such as through gain-of-function mutations in the PCSK9 gene, the protein impairs low-density lipoprotein (LDL)-cholesterol clearance by acting as an antagonist to the LDL receptor, thereby promoting hypercholesterolemia. Conversely, naturally occurring loss-of-function mutations reduce the risk of myocardial infarction by markedly reducing blood cholesterol levels (Chadwick and Musunuru, 2017[24]). Due to this observation, two antibody-based therapies targeting PCSK9 have been developed and recently approved; however, these therapies must be delivered by injection every few weeks (Ito and Santos, 2017[81]). Another example is cardiac amyloidosis, in which production and secretion of a mutant form of the transthyretin (*TTR*) protein by the liver into the bloodstream results in the accumulation of the mutant protein within the cardiac muscle. Disruption of the *TTR *gene in the liver by genome editing might arrest or even reverse the cardiomyopathy, a goal currently being pursued with RNA interference (Coelho et al., 2013[38]).

A distinct approach to leveraging genome editing for the treatment of cardiovascular disorders would be to perform the editing *ex vivo*. This might entail generating induced pluripotent stem cells (iPSCs) from a patient, editing the iPSCs so as to correct a mutation or otherwise render the cells protective against disease, differentiating the edited iPSC into the desired cell type (such as cardiomyocytes), and transplanting the differentiated cells back into the patient (Wu et al., 2018[180]). Even though this approach remains largely theoretical for CVDs. Currently, efforts are directed toward complex, polygenic, chronic cardiac diseases, such as acute heart disease (AHD) and heart failure (HF) (Lim, 2017[112]).

The application of the gene surgery tools in CVDs presents a novel and rapidly advancing technology with promising results. However, significant challenges remain, including enhancing specificity and minimizing off-target effects, increasing efficiency, and improving the selection of targeted sites and delivery methods, and especially for *in vivo* genome engineering. Further refinements are needed to fully exploit the potential of genome editing to be a vital tool for future precision medicine treatment for CVDs.

## Limitations of ZFNs, TALENs and CRISPR/Cas

### Design difficulties and costs 

In spite of the multiple efforts carried out to simplify the gene-editing using ZFNs and TALENs, it still remains difficult, laborious and time-consuming. Scientists are facing more difficulties in building optimized ZFNs with high affinity. In fact, besides the lack of simple ZFN-generation technologies, the positional effects (position of the zing fingers on the protein and the nature of the neighboring fingers) make the ZFN-design more difficult and complex. In addition, the open-source ZFNs have limited target site selection and present several practical limits (Lee et al., 2016[106]; Gupta and Musunuru, 2014[74]; Isalan, 2012[79]). Likewise, since the construction of new TALEN protein is based on the target sequence involving one-to-one recognition rules, it is considered difficult, expensive and time-consuming. On the other hand, the CRISPR/Cas9 system construction is simply based on the alteration of the crRNA sequence of the sgRNA (Chira et al., 2017[34]). 

### Off-target effects 

The ability of both homo or heterodimers of ZFNs and TALENs to recognize the site targets enhances the incidence of off-targets generation (Chandrasegaran and Carroll, 2016[25]; Carlson et al., 2012[20]). It has been demonstrated that the binding affinity of ZFNs is context-dependent. Owing to the presence of resemblance between the target sequence sites and other sites resulting in a high number of targetable sites, ZFNs were shown to possess off-target recognition activities and caused the generation of unknown mutations and cytotoxicity (Li et al., 2013[110]; Pattanayak et al., 2014[138]). Also, it has been found for ZFNs that a low homology of 66 % to the target leads to an off-target cleaving. This resemblance level generating off-target activity was found to be 72 % for TALENs (Fine et al., 2014[57]). A large number of off-target sites of ZFNs in human cells were identified using both *in vitro* and *in silico* approaches (Sander et al., 2013[153]). To avoid cytotoxicity-induced by ZFNs off-targets, commercial ZFNs (custom-made ZFNs) have been developed and were found to be more potent and specific. Unfortunately, the use of custom-made ZFNs in gene editing may be limited due to their high cost (Doudna and Sontheimer, 2014[48]; Koo et al., 2015[98]). Likewise, TALENs have been shown to exhibit several off-targets. At least, three COL7A1, two PPP1R12C, one CCR5 off-targets in humans, and one IgM off-target in Rattus norvegicus were identified for TALENs (Grau et al., 2013[69]). Mussolino et al. (2014[132]) studied the off-target effects of TALENs and ZFNs on three human loci (CCR5, AAVS1, and IL2RG). The results of the bioinformatics-based analysis showed 11 and 4 sites of ZFNs-, and TALENs-associated off-target activity for CCR5 and AAVS1, respectively. 

In the same line, CRISPR-Cas nucleases were demonstrated to induce enhanced rates of off-target mutagenesis in human cells (Fu et al., 2013[60]; Lin et al., 2014[115]). Li et al. (2015[109]) used the Bowtie program to search the off-target-sequence-dependent mutations of both TALENs and CRISPR-Cas in the human genome. Thousands of off-targets sites were found for TALENs, whereas, five off-targets were identified for CRISPR-Cas. In another study using T cells, one off-target site was found for CRISPR/Cas9 in addition to six off-target sites for TALENs (Knipping et al., 2017[95]). Nonetheless, Veres et al. (2014[169]) studied the off-target mutagenesis effects of TALENs and CRISPR-Cas9 in human pluripotent stem cells through whole-genome sequencing. They reported a low incidence of off-target mutations related to TALENs and CRISPR-Cas9. Off-target activity associated with ZFNs, TALENs and CRISPR/Cas may result in DNA damage leading to cytotoxicity, apoptosis and gross chromosomal rearrangements in the human cells (Hendel et al., 2015[77]). Similarly, off-targets mutations induced by CRISPR-Cas9 caused cytotoxicity in other organisms such as Schizosaccharomyces pombe and Saccharomyces cerevisiae (Wang et al., 2016[172]). It has been found that the off-target activity of CRISPR/Cas9 was due to the protospacer sequence in the g-RNA, which explains the fact that Cas9 is less precise in experimental studies when compared to the results obtained via theoretical approaches (Crauciuc et al., 2017[41]). Therefore, due to its important off-target activity, the use of the CRISPR/Cas9 in gene therapy could be controversial (Kruminis-Kaszkiel et al., 2018[99]) and several issues regarding its effectiveness and efficiency should be widely discussed. 

### Efficiency and delivery

To be used in the treatment of multigenic diseases such as cancer, CRISPR/Cas9 system should target different sites on the genome. Constructing CRISPR/Cas9 targeting different loci means the use of multiple sgRNAs and co-transfection of vectors which results in more design complexity and less efficiency *in vitro* and *in vivo* (Chira et al., 2017[34]). In spite of the recent advancements in transfection technologies, it has been found that CRISPR/Cas9 delivery is limited due to the low transfection efficiency and the associated difficulties in transfecting the primary cells (Seki et al., 2018[155]).

One of the biggest challenges of gene therapy is the delivery of the genome editing molecules directly, efficiently and safely to the target cells within their tissues (Carroll, 2016[22]; Cox et al., 2015[40]). The delivery of Cas9 limits the efficiency of gene-editing and therapy (Liang et al., 2015[111]). Actually, the CRISPR/Cas system faces important problems to be safely delivered *in vivo*. Indeed, the spCas9 are characterized by a large molecular size, similar or slightly smaller than that of their adeno-associated virus vectors. Furthermore, the negative charges of RNAs are considered an important obstacle to their diffusion across the cell membrane, besides their degradation induced by the endonucleases (Bayat et al., 2018[13]; Wang et al., 2016[172]).

### Low incidence of homology-directed recombination (HDR) vs high level of NHEJ

The use of CRISPR/Cas9 is characterized by an extremely low incidence of homology-directed repair (HDR) found to be 0.5 – 20 % and important non-homologous end-joining (NHEJ) mechanism reaching 60-100 % in mammalian cells (Lino et al., 2018[116], Li et al., 2017[108]). It has been reported that low HDR hindered the efficiency of the CRISPR/ Cas9 system (Aird et al., 2018[3]). Furthermore, the NHEJ leading to the generation of Indels (insertions/deletions) at the DSBs, resulted in several undesired chromosomal rearrangements and mutations of the target cells (Yanik et al., 2017[185]). When compared to TALEN, Cas9 was shown to possess important NHEJ-inducing activity. Cas9 induced NHEJ 10-fold more than HDR (Miyaoka et al., 2016[129]).

### Ethical and regulatory problems of gene editing 

Recent advances in gene-editing technologies offer promising applications for treating and preventing human diseases, such as Parkinson’s, sickle cell anemia, cardiomyopathy, infectious, and ischemic heart disease. However, many ethical issues have been raised by the use of these technologies in the edition of the human germline. This debate has attracted more attention after the development of CRISPR/Cas9 and its variant methodologies that made genomic editing more accurate and even "easy" compared to older technologies. 

According to UNESCO's International Bioethics Committee (IBC), applications of CRISPR/Cas9 technology should be limited only to the preventative, diagnostic and therapeutic procedures, without altering human embryos. In addition, scientists recognized that CRISPR/Cas9 application in human disease is not entirely safe and effective. An international effort was also initiated to harmonize regulation of the application of genome editing technologies. This effort officially launched in December 2015 with the International Summit on Human Gene Editing and focused on the clinical, ethical, legal and social issues of human gene editing. 

## Future Perspective of Gene Surgery

Despite different therapies and drugs that have been developed for disease treatment, the overall survival rates have not improved much over the last decades. The place of application in CRISPR/Cas9 as a therapeutic approach is astounding. Research has shown that the potential of this gene surgery technology to permanently alter the genetic mutations *in vivo *in the adult liver of mouse models of hereditary genetic disease via homology-directed repair (HDR) pathway, and successfully ameliorating the disorder (Yin et al., 2014[188]). Not only that, the application of CRISPR-based somatic genome editing in mice models and other organisms has greatly accelerated the place of discovery. Perhaps the greatest challenge in the future is the efficiently delivery of CRISPR/Cas9 to the specific targeted cells and reduce the off-target effects (White and Khalili, 2016[176]).

In addition, to enhance its specificity, other crucial limitations need to be overcome before the comprehensive of CRISPR-based genome editing has been realized. For instance, despite available viral vectors have been employed, more effective techniques to deliver the gRNAs and the Cas enzyme to somatic cells of adult animals are needed. Furthermore, it is also important to enhance the efficiency of CRISPR-mediated gene editing for the induction of specific genetic changes such as gain-of-function mutations in oncogenes *in vivo*. This could be achieved by temporarily inhibiting NHEJ to improve HDR (Chu et al., 2015[36]; Maruyama et al., 2015[128]). Although this gene-editing tool has some limitations, further advances of this gene therapy will undoubtedly come rapidly, given the intensity of research efforts in this area.

## Conclusions

In the present review, we summarized the different applications of gene surgery for treating human diseases such as cancer, diabetes, nervous, and cardiovascular diseases, besides the molecular mechanisms involved in these important effects. Recent studies reveal that gene surgery continues to provide promising results regarding their therapeutic effects against retinal diseases, primary immunodeficiencies, neurological disorders, β-thalassemia, hemophilia, diabetes, and cancers. Nonetheless, in spite of the important opportunities both in therapy and translational research and the recent technical advancements, gene surgery still presents some limitations such as the design difficulties and costs regarding ZFNs and TALENs use, off-target effects, low transfection efficiency, *in vivo* delivery-safety and ethical issues. 

## Notes

Bachir Benarba and Adel Gouri contributed equally to the present work.

## Acknowledgements

Adriana Freitas Neves and Thaise Gonçalves de Araujo would like to thank the National Institute of Science and Technology in Theranostics and Nanobiotechnology (INCT-TeraNano-UFU/Brazil).

## Conflict of interest

The authors declare no conflict of interest.

## Figures and Tables

**Table 1:**
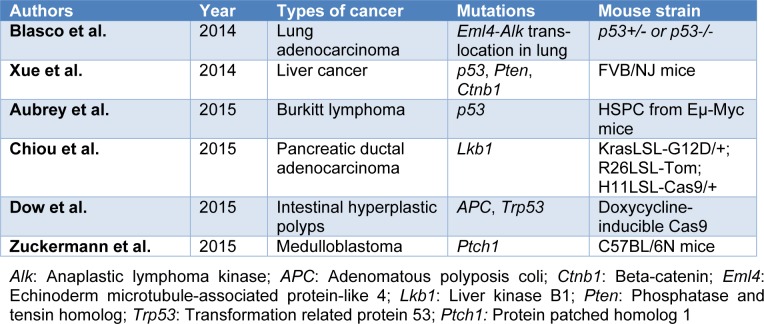
Examples of cancer mouse models developed by CRISPR/Cas9 genome editing
